# NMR-Based Characterization of Wood Decay Fungi as Promising Novel Foods: *Abortiporus biennis*, *Fomitopsis iberica* and *Stereum hirsutum* Mycelia as Case Studies

**DOI:** 10.3390/foods12132507

**Published:** 2023-06-28

**Authors:** Lorenzo Goppa, Mattia Spano, Rebecca Michela Baiguera, Marco Cartabia, Paola Rossi, Luisa Mannina, Elena Savino

**Affiliations:** 1 Department of Earth and Environmental Sciences (DSTA), University of Pavia, Via A. Ferrata 9, 27100 Pavia, Italy; lorenzo.goppa01@universitadipavia.it (L.G.); rebeccamichela.baiguera01@universitadipavia.it (R.M.B.); mc@mogu.bio (M.C.); elena.savino@unipv.it (E.S.); 2 Laboratory of Food Chemistry, Department of Chemistry and Technology of Drugs, Sapienza University of Rome, P.le Aldo Moro 5, 00185 Rome, Italy; luisa.mannina@uniroma1.it; 3 NMR-Based Metabolomics Laboratory (NMLab), Sapienza University of Rome, P.le Aldo Moro 5, 00185 Rome, Italy; 4 MOGU S.r.l., Via S. Francesco d’Assisi 4, 21020 Inarzo, Italy; 5 Department of Biology and Biotechnology “L. Spallanzani”, University of Pavia, 27100 Pavia, Italy; paola.rossi@unipv.it

**Keywords:** *Abortiporus biennis*, *Fomitopsis iberica*, *Stereum hirsutum*, mycelia, NMR, metabolomic, Novel Food

## Abstract

Wood Decay Fungi (WDF) are fungi specialized in degrading wood. An interesting perspective is their use as a source of Novel Foods or food ingredients. Here, for the first time, the metabolite profiling of hydroalcoholic and organic extracts from *A. biennis*, *F. iberica*, *S. hirsutum* mycelia was investigated by NMR methodology. Amino acids (alanine, arginine, asparagine, aspartate, betaine, GABA, glutamate, glutamine, histidine, isoleucine, leucine, lysine, phenylalanine, threonine, tryptophan, tyrosine, valine), sugars (galactose, glucose, maltose, trehalose, mannitol), organic acids (acetate, citrate, formate, fumarate, lactate, malate, succinate), adenosine, choline, uracil and uridine were identified and quantified in the hydroalcoholic extracts, whereas the ^1^H spectra of organic extracts showed the presence of saturated, mono-unsaturated and di-unsaturated fatty chains, ergosterol,1,2-diacyl-*sn*-glycero-3-phosphatidylethanolamine, and 1,2-diacyl-*sas*glycero-3-phosphatidylcholine. *A. biennis* extracts showed the highest amino acid concentration. Some compounds were detected only in specific species: betaine and mannitol in *S. hirsutum*, maltose in *A. biennis*, galactose in *F. iberica*, GABA in *F. iberica* and *S. hirsutum*, and acetate in *A. biennis* and *S. hirsutum*. *S. hirsutum* showed the highest saturated fatty chain concentration, whereas DUFA reached the highest concentration in *A. biennis.* A high amount of ergosterol was measured both in *A. biennis* and *F. iberica*. The reported results can be useful in the development of WDF-based products with a high nutritional and nutraceutical value.

## 1. Introduction

Wood Decay Fungi (WDF) are a group of fungi able to grow on different forms of wood substrates such as living plants, dying trees, fallen wood or dead wood [1]. Due to their rich enzymatic pools, WDF specifically degrade wood lignin, cellulose, hemicelluloses, and pectins, becoming useful in many applicative fields such as degradation of organic pollutants, bioremediation, bioadsorption, and bioaccumulation of metal ions in living or dead biomass [2]. An interesting perspective is the possible use of WDF as source of nutraceuticals or Novel Foods.

Recently, several mushroom ingredients or mycelia powders have been studied, tested and approved as Novel Foods. It is the case of the chitin–glucan from *Aspergillus niger* Tiegh. and *Fomes fomentarius* (L.) Fr. [3], the recently approved vitamin D_2_ from *Agaricus bisporus* (J.E. Lange) Imbach powder [4] or the dehydrated mycelia powder from different WDF species including *Ganoderma lucidum* (Curtis) P. Karst., *Grifola frondosa* (Dicks.) Gray, *Hericium erinaceus* (Bull.) Pers., *Lentinula edodes* (Berk.) Pegler, *Pleurotus eryngii* (DC.) Quèl., *Pleurotus ostreatus* (Jacq.) P. Kumm., and *Polyporus umbellatus* (Pers.) Fr. [5].

Regarding WDF, only the mycelia of a few species such as *G. lucidum* or *G. frondosa* have been extensively investigated because of their relevance in the medical field. In particular, *G. lucidum* has turned out to be rich in phenolics, polysaccharides, and triterpenoids, responsible for both immunomodulatory and probiotic activities [6], whereas *G. frondosa*, rich in amino acids, sugars, peptides, and polysaccharides has shown antimicrobial, antidiabetic, probiotic, antioxidant, lipid metabolism regulation, and anti-hypertensive activities [7].

Other WDF species of potential interest in the nutraceutical and Novel Food fields are *Abortiporus biennis* (Bull.) Singer, *Fomitopsis iberica* Melo, Ryvarden, and *Stereum hirsutum* (Willd.) Pers. Up to now, the chemical analysis of these three species has been focused mainly on the determination of polysaccharides content, namely alpha-glucans and beta-glucans in *A. biennis* and *F. iberica*, respectively, and chitin in *S. hirsutum* [2,8]. *S. hirsutum* mycelium has also been investigated through GC-TOF-MS [9] analysis, showing the presence of sugars (meso-erythritol, ribose, glucose, 6-deoxyglucose, trehalose), organic acids (2-hydroxyhexanoic acid, 2,3-dihydroxybutanoic acid, citramalic acid, 2-oxoglutaric acid), and amino acids (glycine, alanine, threonine, glutamate).

As widely reported [10,11], NMR untargeted approach is one of the main powerful methodologies useful to achieve the metabolite profiling of complex biological matrices and to carry out quantitative comparison among different vegetable varieties [12,13].

Although NMR spectroscopy is characterized by a lower sensitivity with respect to other methodologies such as Mass Spectroscopy (alone or coupled with separation systems), it provides a sure structural determination since it is confirmed by ^1^H spectrum features (chemical shift, *J* coupling constants, multiplicity) and two-dimensional experiments. In the context of first-time characterization of the metabolite profile of a certain matrix, this approach is very useful to offer concrete information.

In this paper, a detailed NMR investigation of the hydroalcoholic and organic *A. biennis*, *F. iberica* and *S. hirsutum* mycelia extracts was carried out to obtain their complete metabolites profile as the first step in the development of WDF-based Novel Foods or food ingredients. The identified metabolites were quantified to determine strengths and weaknesses of each investigated species, thus suggesting potential food applications for the analyzed species.

## 2. Materials and Methods

### 2.1. Fungal Strains

The Wild Type (WT) sporophores of *A. biennis*, *F. iberica* and *S. hirsutum* were collected in Italy and strains were isolated in pure culture. As reported in [14], all the strains were identified both by macro- and micro-morphological cultural characteristics and by molecular analysis of the Internal Transcribed Spacer (ITS) region. The three analyzed strains belong both to the Fungal Research Culture Collection (MicUNIPV) of the Mycology Laboratory at the Department of Earth and Environmental Sciences (DSTA) (University of Pavia, Italy) and MOGU’s fungal strain collection (MFSC). The strains, which, respectively, have the following codes: MicUNIPV A.b.6, MicUNIPV F.b.1, MicUNIPV S.h.1, and MFSC 064-18, MFSC 104-19, MFSC 073-18, were maintained through different cultural media and preserved at −80 °C in MicUNIPV.

### 2.2. Mycelia Samples Preparation

Fungal strains were first grown up to 15 days at 25 °C in Petri dishes with a 2%*_w/v_* Malt Extract Agar (MEA) to standardize the inoculum conditions and reactivity. Ten colonized portions of MEA (about 0.125 cm^3^ each) were sterilely inoculated into flasks (capacity of 1 L) containing a 2%*_w/v_* ME previously sterilized by autoclave (121 °C, 20 min) and corked by raw cotton to favor gaseous exchange. Incubation was carried out in dark and static condition at 25 °C. After 15 days, each mycelium was gently washed with deionized water and lyophilized for 24 h at −50 °C and 1 mbar. Mycelia were stored in a freezer at −20 °C.

### 2.3. Samples Extraction

Extraction of both hydroalcoholic and organic fractions was carried out using the Bligh–Dyer protocol by modifying a previously described procedure [15]. A 100 mg aliquot of dried and pulverized sample was added to 3 mL of a CH_3_OH/CHCl_3_ 2:1 *v*/*v* mixture and 0.8 mL of distillated H_2_O, followed by sonication. Afterwards, 1 mL of CHCl_3_ and 1 mL of distillated H_2_O were added to the system that was finally centrifugated, allowing the separation of hydroalcoholic and organic phases. Extraction was repeated two more times on residual pellet and the reunited hydroalcoholic and organic phases were dried with an N_2_ flux.

### 2.4. NMR Analysis

NMR analyses were carried out on a 600 MHz spectrometer (Jeol JNM-ECZ 600R) equipped with a 5 mm FG/RO DIGITAL AUTOTUNE probe.

**Figure 1:**
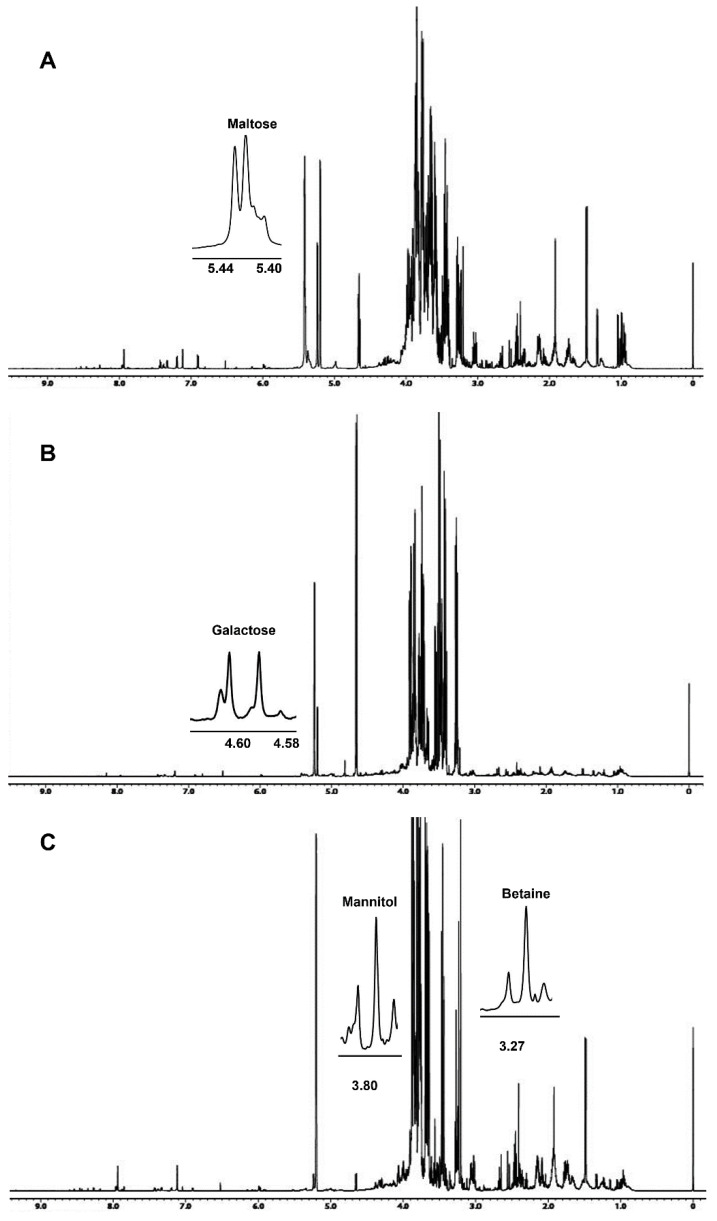
The 600.13 MHz ^1^H NMR spectra of Bligh–Dyer hydroalcoholic extracts of (**A**) *A. biennis*, (**B**) *F. iberica* and (**C**) *S. hirsutum* mycelia. Signals characteristic of the metabolites detected only in the corresponding species are expanded in the spectra.

Dried hydroalcoholic phases were dissolved in 700 μL of a 100 mM phosphate buffer/D_2_O, containing a 0.5 mM TSP (3-(trimethylsilyl)propionic acid sodium salt) as internal standard. ^1^H spectra, Figure 1, were obtained at 298 K using the following parameters: 128 scans, residual HDO signal suppression with a pre-saturation pulse, a 7.7 s relaxation delay, a 90° pulse of 8.3 μs, 64 k data points, and a 9000 Hz spectral width. ^1^H spectra were referenced to a TSP methyl group signal in D_2_O (δH = 0.00 ppm).

Dried organic phases were dissolved in 700 μL of a CDCl_3_/CD_3_OD 2:1 *v*/*v* mixture. ^1^H spectra were obtained at 298 K using the following parameters: 128 scans, a 7.7 s relaxation delay, a 90° pulse of 8.3 μs, 64 k data points, and a 9000 Hz spectral width. ^1^H spectra were referenced to a CHD_2_ residual signal of methanol (δH = 3.34 ppm).

Two-dimensional NMR experiments, namely ^1^H-^1^H TOCSY, ^1^H-^13^C HSQC, and ^1^H-^13^C HMBC, were carried out on hydroalcoholic extracts. In particular, ^1^H-^1^H TOCSY were acquired with 56 scans, 8 k data points in *f*_2_ and 128 in *f*_1_, a 50 ms mixing time, a 2 s relaxation delay, and a 9000 Hz spectral width in both dimensions. The ^1^H-^13^C HSQC experiments were acquired with 88 scans, 8 k data points in *f*_2_ and 256 in *f*_1_, a 3 s relaxation delay, and a spectral width of 9000 Hz and 33,000 Hz for *f*_2_ and *f*_1_, respectively. The ^1^H-^13^C HMBC experiments were acquired with 84 scans, 8 k data points in *f*_2_ and 165 in *f*_1_, a 2 s relaxation delay, and a spectral width of 9000 Hz and 37,500 Hz for *f*_2_ and *f*_1_, respectively Spectrum processing and signal integration were carried out with the JEOL Delta software (v5.3.1).

To quantify the identified metabolites in the hydroalcoholic extracts, the integrals of the corresponding selected ^1^H resonances were measured with respect to TSP. Three replicates were made, and the results were expressed as mg/100 g of sample ± SD.

To quantify the identified metabolites in the organic extracts, integrals of the corresponding selected ^1^H resonances were measured and expressed as molar % ± SD, on three replicates, by applying the following equations:

%ERG = 100 × (2I_ERG_/I_tot_),

%DUFA = 100 × (I_DUFA_/I_tot_),

%MUFA = 100 × (I_TOT UFA_ − 2I_DUFA_)/I_tot_,

%TOT FA = 100 × (I_TOT FA_/I_tot_),

%TOT UFA = %MUFA + %DUFA,

%TOT SFA = %TOT FA − %TOT UFA,

%PC = 100 × (4I_PC_/9I_tot_),

%PE = 100 × (2I_PE_/I_tot_).

I_ERG_, I_DUFA_, I_TOT UFA_, I_TOT FA_, I_PC_, and I_PE_ are the integral values of ergosterol, di-unsaturated fatty acids, mono-unsaturated fatty acids, total fatty acids, total unsaturated fatty acids, total saturated fatty acids, phosphatidylcholine, and phosphatidylethanolamine signals, respectively; see Table 1. In particular, to integrate TOT UFA, signals in the range of 5.33–5.35 ppm were considered, corresponding to double-bound protons. To integrate TOT FA, signals in the range of 2.28–2.30 were considered, corresponding to α-CH_2_ groups of all fatty acids.

I_tot_ is obtained by the following equation:

I_tot_ = I_TOT FA_ + 2I_ERG_.

%ERG, %DUFA, %MUFA, %TOT FA, %TOT UFA, %TOT SFA, %PC, and %PE are the molar % of ergosterol, di-unsaturated fatty acids, mono-unsaturated fatty acids, total fatty acids, total unsaturated fatty acids, total saturated fatty acids, phosphatidylcholine, and phosphatidylethanolamine, respectively.

## 3. Results and Discussion

### 3.1. NMR Assignment of Bligh–Dyer Extracts

The ^1^H NMR spectra of *A*. *biennis*, *F. iberica* and *S. hirsutum* hydroalcoholic extracts are reported in Figure 1. Spectral assignments reported in Table 1 were obtained by means of 2D experiments and literature data relative to other vegetal matrices analyzed in the same experimental conditions [16,17].

The ^1^H-^1^H TOCSY was useful to confirm some assignments or to solve dubious cases. For instance, the ^1^H spectrum showed the presence of two doublets at 1.33 ppm and 1.34 ppm characterized by the same *J* coupling constant of 6.6 Hz, typically due to terminal CH_3_. The doublet at 1.33 ppm was assigned to CH_3_ of lactate, showing in the TOCSY map a typical spin correlation with the α-CH at 4.13, whereas the doublet at 1.34 ppm was assigned to threonine, showing a correlation with α-CH and β-CH at 3.58 and 4.27 ppm, respectively.

In some cases, due to strong signal overlapping, the addition of standard compounds was necessary to confirm metabolite assignment. It is the case of betaine and mannitol of *S. hirsutum* hydroalcoholic extracts, whose signals at 3.27 ppm (betaine) and 3.68, 3.77, 3.80, 3.87 ppm (mannitol) showed an increase in intensity after the standard addition.

The ^1^H spectra of organic extracts were assigned by literature data [18] showing the presence of saturated fatty acids, mono-unsaturated fatty acids, di-unsaturated fatty acids, ergosterol, 1,2-diacyl-*sn*-glycero-3-phosphatidylethanolamine, and 1,2-diacyl-*sn*-glycero-3-phosphatidylcholine.

### 3.2. Quantitative Metabolite Profile: Comparison between A. biennis, F. iberica and S. hirsutum Mycelia

The metabolites identified in the hydroalcoholic and organic extracts were quantified according to the procedure reported in Experimental section. Data are reported as histograms and discussed separately according to the class of compounds.

#### 3.2.1. Amino Acids

It is noteworthy that no chemical or enzymatic protein hydrolysis was carried out before the NMR analysis, so the here-discussed amino acids (AAs)are referred to as the free ones, naturally present in the samples.

Seventeen amino acids were identified in the ^1^H spectra of hydroalcoholic extracts.

**Figure 2:**
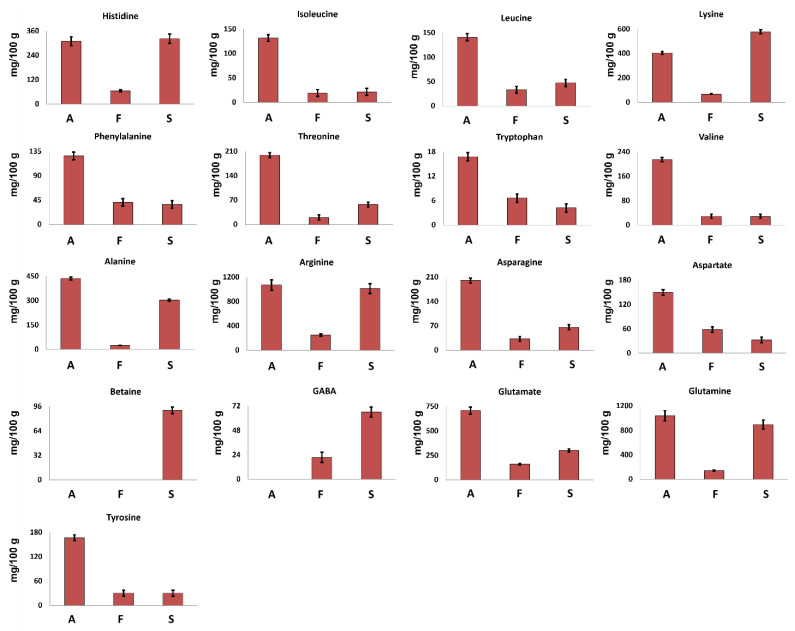
Quantitative histograms relative to free amino acids measured in ^1^H NMR spectra of Bligh–Dyer hydroalcoholic extracts. *A. biennis* (A), *F. iberica* (F), *S. hirsutum* (S).

Betaine, a non-protein amino acid, was detected only in *S. hirsutum* mycelium (see Figure 1) at a concentration of 91 mg/100 g (Figure 2), whereas GABA was detected in *S. hirsutum* as well as *F. iberica* but not in *A. biennis* mycelium.

The other 15 amino acids were detected in all the investigated samples. Arginine was the most abundant amino acid, whereas tryptophan was present at the lowest concentration.

*A. biennis* was characterized by the highest concentration of all AAs, except for lysine, suggesting this species as a significative AAs source.

All the investigated samples showed the presence of all the essential amino acids (EAAs) except methionine, confirming their nutritional value. In *A. biennis* and *S. hirsutum* in particular, EAAs represent more than 35% of the total free AAs, whereas in *F. iberica*, EAAs represent less than 30%.

In all the investigated samples according to literature data [19], lysine turned out to be the most abundant EAA, reaching a concentration of 404 mg/100 g and 579 mg/100 g in *A. biennis* and *S. hirsutum*, respectively.

From a quantitative point of view, glutamine and glutamate were found to be the most abundant amino acids in *A. biennis*, followed by alanine and lysine. In particular, the high concentration of glutamate was expected, since it has been found to be the main non-essential amino acid in several mushrooms, playing a role as a precursor for the synthesis of other amino acids. Moreover, the umami taste that is common in certain kinds of foods, including mushrooms, can be linked to the presence of glutamic acid [20].

Regarding *S. hirsutum*, comparing the results obtained here with those of a previously reported study [9], an improvement of the amino acids profile was achieved. In the cited paper [9], just four amino acids have been identified (glycine, alanine, threonine, glutamate), whereas in this work, seventeen molecules of this class were identified. In any case, glycine reported by Peiris et al. [9] was not detected in this work.

In Table 2, AAs content of some species proposed/approved as Novel Foods (*Agaricus blazei* Murril, *G. frondosa*, *L. edodes*) [21,22,23] is reported together with AAs content of *A. biennis* species determined here. Due to the different extraction procedures and the different analytical methods, it is not possible to carry out a direct quantitative comparison. However, it is possible to observe interesting trends regarding the AAs profile of the different species.

The four species are characterized by different amino acid profiles. Among the essential amino acids, methionine was not detected in *A. biennis*. On the other hand, tryptophan was identified and quantified in *A. biennis* and not detected in the other three species. Regarding the non-essential amino acids, asparagine and glutamine were detected only in *A. biennis*, whereas arginine and serine were detected only in the other three species.

In order to understand whether the AAs content of *A. biennis* can be considered advantageous in comparison with other non-fungal food, a comparison between its AAs content and the ones of some vegetables is reported here; see Table 3. Considering the classification of cereals and grain products, starchy roots and tubers, dry legumes, nuts and seeds, vegetables and, finally, fruit offered by the Food and Agriculture Organization of United Nations (FAO) [24], in order to make comparison clearer, only one representative food from each category was taken into account.

Eggs, milk, meat and fish were excluded from the comparison because they were not in compliance with the purpose of the work.

Although the data reported in Table 3 cannot be considered representative of all the non-fungal foods listed in the FAO document, the comparison of the indicated values can be useful for making some considerations. In particular, *A. biennis* mycelium showed the highest concentration of asparagine and glutamate and was proven to be second after lentils in regard to histidine and lysine (both EAAs) concentrations.

#### 3.2.2. Sugars

Glucose and trehalose were identified and quantified in all the investigated mycelia samples. Trehalose, a typical sugar of mushrooms, is a low glycemic disaccharide able to lower the postprandial glycaemia and to induce biogenesis of lysosomes and autophagosomes [25].

**Figure 3:**
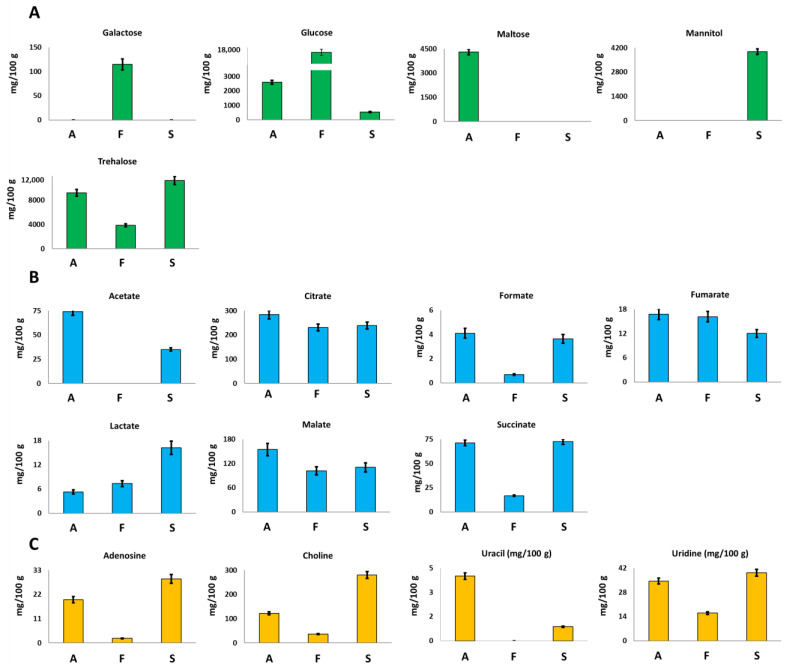
Quantitative histograms relative to (**A**) free sugars, (**B**) organic acids, and (**C**) Adenosine, Choline, Uracil and Uridine quantified in NMR analysis of Bligh–Dyer hydroalcoholic extracts. *A. biennis* (A), *F. iberica* (F), *S. hirsutum* (S).

*F. iberica* showed the highest amount of glucose, 16,491 mg/100 g, about 6 times and 30 times higher than the amount detected in *A. biennis* and *S. hirsutum*, respectively; see Figure 3A.

In *A. biennis* and *S. hirsutum*, trehalose turned out to be the most abundant sugar. In particular, among the three species, *S. hirsutum* showed the highest content, whereas *F. iberica* displayed the lowest one.

Interestingly, other sugars were detected only in the specific species: galactose was detected only in *F. iberica*, maltose was detected in *A. biennis*, and mannitol was detected in *S. hirsutum*, which offers the potential role of species markers to these metabolites.

Also in the case of sugars, the comparison between the results for *S. hirsutum* reported here and those of Peiris et al. [9] underlined the presence of qualitative differences. In particular, glucose and trehalose were detected in both studies, whereas meso-erythritol, ribose, and 6-deoxyglucose were not detected here. This difference could be due to the lower sensitivity of NMR spectroscopy in respect to GC-TOF-MS, or to the used strain, whereas a difference due to the growth medium is excluded since both studies were carried out using the same medium (Malt extract). On the contrary, mannitol was reported here.

#### 3.2.3. Organic Acids

Mushrooms are well known and largely used for the production of organic acids in food, pharmaceutical, and technical sectors [26]. The samples investigated here showed the presence of seven organic acids, namely acetate, citrate, formate, fumarate, lactate, malate, and succinate. Among them, citrate turned out to be present in the highest concentration in the three species. In particular, *A. biennis* species showed the highest concentration of citrate (Figure 3B), malate, formate, fumarate, and acetate. Acetate was not detected in *F. iberica*. Lactate concentration turned out to be at least three times higher in *S. hirsutum* than in the other two species. The obtained qualitative data are in accordance with the literature data regarding the organic acids profile of edible mushrooms [27,28]. In particular, except for acetate, the metabolites detected here have been already identified in other fungal species. Moreover, it is noteworthy that it was not possible to verify the presence of oxalate, a dicarboxylic acid typical of fungal species, since it consists of two bounded carboxylic groups whose signals cannot be detected in the ^1^H NMR spectrum of aqueous samples.

In the case of organic acids identified in *S. hirsutum*, the comparison with Peiris et al. [9] underlined a completely different qualitative profile, with 2-hydroxyhexanoic acid, 2,3-dihydroxybutanoic acid, citramalic acid, and 2-oxoglutaric acid identified in the cited paper but not in the present work.

#### 3.2.4. Other Compounds

Adenosine, choline, and uridine were present at the highest concentration in *S. hirsutum*, whereas the highest amount of uracil was measured in *A. biennis*, Figure 3C. This last metabolite was not detected in *F. iberica*.

Choline, or Vitamin J, is an amine only partially synthesized by the human body (due to the presence of cobalamin and folic acid); therefore, its supply should be ensured, above all, through food. This metabolite is important for the synthesis of phospholipids in cell membranes, methyl metabolism, acetylcholine synthesis, and cholinergic neurotransmission in humans [29].

According to the National Academy of Sciences, USA, foods with the highest choline concentrations are beef liver, chicken liver and eggs [30]. No data are reported in the literature about choline determination on the mushrooms considered here.

Considering that the recommended dietary daily intake of choline is 550 mg of total choline per day for men and 425 mg per day for women, *S. hirsutum* may represent an interesting dietary source for this nutrient. This is reinforced by the presence of betaine (not detected in *A. biennis* and *F. iberica*), that is a choline metabolite that cannot be converted to choline but can be used as a methyl donor, sparing some choline requirements [31].

#### 3.2.5. Apolar Fraction

**Figure 4:**
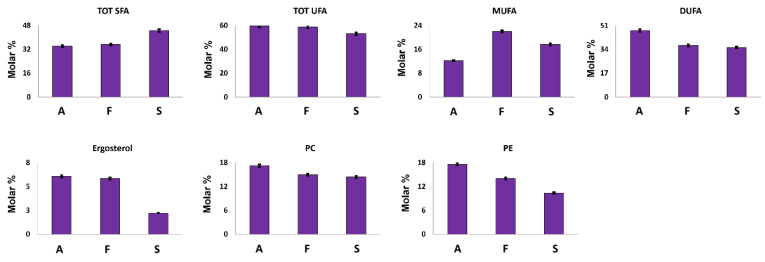
Quantitative histograms relative to quantified fatty chains, ergosterol and polar lipid heads from the NMR analysis of Bligh–Dyer hydroalcoholic extracts. *A. biennis* (A), *F. iberica* (F), *S. hirsutum* (S).

Ergosterol is a sterol present in the cell membrane of mushrooms where it exerts a similar function to those of cholesterol in animal cells. It can also be considered an important nutritional compound since it is the precursor of vitamin D [32]. As expected, ergosterol was identified in all the investigated samples; its highest concentration was measured in *A. biennis* and *F. iberica* extracts (Figure 4).

Fatty chains represent the major compounds of Bligh–Dyer organic extracts. In *A. biennis* and *F. iberica* samples, unsaturated fatty chains (UFA) showed an average molar concentration of 59% (Figure 4). In particular, *A. biennis* was characterized by the highest DUFA and the lowest MUFA concentrations. Among them, DUFA were present in high concentration and can be attributed to ω-6 linoleic acid, an essential fatty acid strongly recommended in the human diet, and whose presence in mushrooms has been largely demonstrated [33,34,35].

It is noteworthy that tri-unsaturated fatty chains were not detected in the ^1^H NMR spectra of the investigated samples, thus suggesting the absence, or at least a non-NMR relievable concentration, of this essential fatty acid. These data are strongly supported by the literature data, where tri-unsaturated fatty acids in mushrooms have shown to be absent or present in very low concentrations [33,34,35,36].

## 4. Conclusions

In this study, the NMR-based chemical profile of three fungal mycelia, namely *A. biennis*, *F. iberica* and *S. hirsutum*, was reported, each species characterized by a peculiar chemical profile and thus by a potential nutritional value. The overall chemical profile can represent an important first step towards the potential use of WDF mycelia as food matrices or food ingredients. New products with nutritional and nutraceutical properties could be developed as a mixture of different species, opening new perspective in WDF sectors.

## Figures and Tables

**Table 1:** Metabolites identified in the 600.17 MHz ^1^H NMR spectra of WDG Bligh–Dyer hydroalcoholic extracts dissolved in 100 mM phosphate buffer/D_2_O containing TSP 0.5 mM and Bligh–Dyer organic extracts dissolved in CDCl_3_/MeOD 2:1 *v*/*v* solution.

Metabolite	Assignment	^1^H (ppm)	Multiplicity [*J* (Hz)]	^13^C (ppm)
***Hydroalcoholic extract***				
*Amino acids and derivatives*				
Alanine ^a,b,c^	COO-			177.0
	α-CH	3.80		51.6
	β-CH_3_	1.49 *	d [7.3]	17.2
Arginine ^a,b,c^	α-CH	3.76		
	β-CH_2_	1.93		
	γ-CH	1.66 *	m	25.0
	γ′-CH	1.74	m	25.0
	δ-CH_3_	3.25		
Asparagine ^a,b,c^	α-CH	4.02		
	β, β′-CH_2_	2.87; 2.95 *	dd [7.4; 16.9]	
Aspartate ^a,b,c^	α-CH	3.92		
	β, β′-CH_2_	2.72; 2.81 *	dd [17.4;3.8]	
Betaine ^c^	N(CH_3_)_3_^+^	3.27 *	s	
GABA ^b,c^	α-CH_2_	2.30 *	t [7.4]	35.4
	β-CH_2_	1.94		24.3
	γ-CH_2_	3.02		39.8
Glutamate ^a,b,c^	α-CH	3.78		55.6
	β, β′-CH_2_	2.07;2.14		28.0
	γ-CH_2_	2.36 *	m	34.8
Glutamine ^a,b,c^	α-CH	3.78		
	β, β′-CH_2_	2.15		
	γ-CH_2_	2.46 *	m	
Histidine ^a,b,c^	CH-3, ring	7.94	s	
	CH-5, ring	7.12 *	s	
Isoleucine ^a,b,c^	α-CH	3.69		60.7
	β-CH	1.99		37.1
	γ-CH_3_	1.02 *	d [7.1]	15.8
	γ′-CH	1.27		25.9
	δ-CH_3_	0.94	t [7.4]	12.3
Leucine ^a,b,c^	α-CH	3.74		
	β-CH_2_	1.72		40.8
	δ, δ′-CH_3_	0.96; 0.97 *	d [6.2]	25.7
Lysine ^a,b,c^	α-CH	3.77		
	β-CH_2_	1.93		
	γ-CH_2_	1.49		22.7
	δ-CH_2_	1.74		27.7
	ε-CH_2_	3.03 *	t [7.3]	40.2
Phenylalanine ^a,b,c^	CH-2,6 ring	7.34	m	
	CH-3,5 ring	7.43 *	m	
	CH-4 ring	7.38	m	
Threonine ^a,b,c^	α-CH	3.58		61.7
	β-CH	4.27		67.0
	γ-CH_3_	1.34 *	d [6.6]	21.2
Tryptophan ^a,b,c^	CH-4, ring	7.74	d [8.1]	
	CH-5, ring	7.21		
	CH-6, ring	7.29		
	CH-7, ring	7.55 *	d [8.1]	
Tyrosine ^a,b,c^	CH-2,6 ring	6.90 *	d [8.6]	116.9
	CH-3,5 ring	7.20	d [8.6]	129.5
Valine ^a,b,c^	α-CH	3.63		61.8
	β-CH	2.29		30.3
	γ-CH_3_	0.99	d [7.06]	18.0
	γ′-CH_3_	1.05 *	d [7.06]	19.2
*Sugars and polyols*				
β-Galactose ^b^	CH-1	4.60 *	d [7.9]	
	CH-2	3.51		
	CH-3	3.67		
α-Glucose ^a,b,c^	CH-1	5.24	d [3.8]	
	CH-2	3.56		72.7
	CH-3	3.74		74.2
	CH-4	3.42		71.3
	CH-5	3.84		
β-Glucose ^a,b,c^	CH-1	4.65 *	d [7.8]	97.0
	CH-2	3.28		75.5
	CH-3	3.50		76.9
	CH-4	3.43		
α-Maltose ^a^	CH-1	5.24	d [3.6]	
	CH-2	3.57		72.7
	CH-3	3.74		74.2
	CH-4	3.45		71.3
	CH-5	3.88		72.5
	CH_2_-6	3.98		60.1
	CH-1′	5.42 *	d [3.8]	
	CH-2′	3.62		72.7
	CH-3′	3.70		73.9
	CH-4′			78.0
β-Maltose ^a^	CH-1	4.67	d [7.8]	97.4
	CH-2	3.30		72.7
	CH-3	3.79		74.2
	CH-4	3.65		71.3
	CH-1′	5.42 *	d [3.8]	
	CH-2′	3.62		72.7
	CH-3′	3.70		73.9
	CH-4′			78.0
Mannitol ^c^	CH-1,6	3.68	dd [6.2; 11.9]	
	CH-1′,6′	3.87	dd [2.9; 11.9]	
	CH-2,5	3.77	m	
	CH-3,4	3.80 *	m	
Trehalose ^a,b,c^	CH-1	5.20 *	d [3.8]	
	CH-2	3.64		
	CH-3	3.86		
	CH-4	3.46		
*Organic acids*				
Acetate ^a,c^	COO^-^			182.9
	α-CH_3_	1.92 *	s	24.4
Citrate ^a,b,c^	α, γ-CH	2.55 *	d [15.9]	46.9
	α′, γ′-CH	2.66	d [15.9]	46.9
	β-C			76.5
	1,5-COO^-^			180.3
	6-COO^-^			183.2
Formate ^a,b,c^	HCOO^-^	8.46 *	s	
Fumarate ^a,b,c^	α, β-CH=CH	6.53 *	s	
Lactate ^a,b,c^	α-CH	4.13		
	β-CH_3_	1.33 *	d [6.6]	
Malate ^a,b,c^	α-CH	4.30 *	dd [9.8; 3.2]	
	β-CH	2.67	dd [15.6; 3.2]	
	β′-CH	2.39	dd [15.6; 9.8]	
Succinate ^a,b,c^	α, β-CH_2_	2.41 *	s	
*Other metabolites*				
Adenosine ^a,b,c^	CH-2	8.36 *	s	
	CH-8	8.27	s	
	CH-1′	6.08	d [6.2]	
Choline ^a,b,c^	N(CH_3_)_3_^+^	3.21 *	s	55.2
	α-CH_2_			68.7
Uridine ^a,b,c^	CH-5	5.90	d [8.1]	
	CH-6	7.88 *	d [8.1]	
	CH-1′	5.92	d [4.5]	
Uracil ^a,c^	CH-5	5.80 *	d [8.1]	
	CH-6	7.54	d [8.1]	
***Organic extract***				
Mono-unsaturated fatty chain ^a,b,c^	COO			173.9
(Cn:1 Δ^9^)	CH_2_-2	2.28 *		34.6
	CH_2_-3	1.57	m	25.4
	CH_2_-4,7	1.30	m	29.5
	CH_2_-8	2.01	m	27.6
	CH=CH 9,10	5.33 *	m	130.4
	CH_2_-11	2.01	m	27.6
	CH_2_	1.33–1.28	m	29.8–32.0
	CH_2_-n-1	1.26	m	22.9
	CH_3_-n	0.87	t	14.2
Di-unsaturated fatty chain ^a,b,c^	COO			173.9
(Cn:2 Δ^9,12^)	CH_2_-2	2.30 *		34.2
	CH_2_-3	1.57	m	25.3
	CH_2_-4,7	1.32–1.28	m	29.8
	CH_2_-8	2.09	m	27.2
	CH=9	5.35 *	m	130.4
	CH=10	5.33 *	m	128.2
	CH_2_-11	2.77 *	t [6.7]	26.0
	CH=12	5.33 *	m	128.2
	CH=13	5.35 *	m	130.4
	CH_2_-14	2.09	m	27.2
	CH_2_	1.26–1.27	m	29.8–32.0
	CH_2_-n-1	1.23	m	22.5
	CH_3_-n	0.89	t [6.4]	14.2
Di-unsaturated fatty chain ^a,b,c^	COO			173.9
(Cn:2 Δ^9,12^)	CH_2_-2	2.30 *		34.2
	CH_2_-3	1.57	m	25.3
	CH_2_-4,7	1.32–1.28	m	29.8
	CH_2_-8	2.09	m	27.2
	CH=9	5.35 *	m	130.4
	CH=10	5.33 *	m	128.2
	CH_2_-11	2.77 *	t [6.7]	26.0
	CH=12	5.33 *	m	128.2
	CH=13	5.35 *	m	130.4
	CH_2_-14	2.09	m	27.2
	CH_2_	1.26–1.27	m	29.8–32.0
	CH_2_-n-1	1.23	m	22.5
	CH_3_-n	0.89	t [6.4]	14.2
Saturated fatty acids ^a,b,c^	COO			173.9
	CH_2_-2	2.28 *		34.6
	CH_2_-3	1.57	m	25.4
	CH_2_	1.28–1.22	m	29.8–32.0
	CH_2_ n-1	1.25		22.5
	CH_3_ n	0.87	t	14.2
Ergosterol ^a,b,c^	CH=6	5.55 *		
	CH=7	5.43		
	CH_2_-12			41.6
	C-13			45.7
	CH-14			58.6
	CH-17			57.0
	CH_3_-18	0.66	s	12.4
	CH=22,23	5.27	m	
1,2-Diacyl-*sn*-glycero-3-phosphatidylethanolamine ^a,b,c^	CH_2_N	3.10 *	t [5.0]	
	CH_2_OP	4.08		
	CH_2_ *sn1*	4.45; 4.16		62.2
	CH *sn2*	5.28		69.1
	CH_2_ *sn3*	4.05		64.7
1,2-Diacyl-*sn*-glycero-3-phosphatidylcholine ^a,b,c^	^+^N(CH_3_)_3_	3.21 *	s	54.5
	CH_2_N^+^	3.64		66.7
	CH_2_OP	4.31		
	CH_2_ *sn1*	4.45; 4.16		62.2
	CH *sn2*	5.28		69.1
	CH_2_ *sn3*	4.05		64.7

Asterisks (*) indicate signals selected for integration; ^a^ metabolite identified in *A*. *biennis*; ^b^ metabolite identified in *F. iberica*; ^c^ metabolite identified in *S. hirsutum*.

**Table 2:** AAs concentrations in *A. biennis* mycelium compared to the ones of other mushroom mycelia proposed/approved as Novel Foods.

Amino Acid	*A. biennis* (mg/g)	*A. blazei* Chang et al., 2001 [21] (mg/g)	*G. frondosa* Tsai et al., 2006 [22] (mg/g)	*L. edodes* Aminuddin et al., 2007 [23] (mg/g)
Leucine	1.41 ± 0.02	0.31 ± < 0.1	4.92 ± 0.04	0.83 ± 0.01
Isoleucine	1.32 ± 0.02	0.21 ± < 0.1	2.80 ± 0.02	0.66 ± 0.04
Valine	2.15 ± 0.03	1.76 ± 0.07	4.13 ± 0.11	0.62 ± 0.01
Histidine	3.08 ± 0.06	0.66 ± 0.28	4.1 ± 0.17	0.26 ± 0.02
Lysine	4.04 ± 0.45	0.61 ± < 0.1	0.22 ± 0.01	0.90 ± 0.03
Methionine	ND ^1^	0.67 ± 0.27	2.67 ± 0.10	0.06 ± 0.01
Phenylalanine	1.27 ± 0.03	0.17 ± < 0.1	1.66 ± 0.42	0.61 ± 0.01
Threonine	1.99 ± 0.07	0.53 ± 0.03	8.23 ± 0.36	0.58 ± 0.02
Tryptophan	0.16 ± 0.03	ND	ND	ND
Alanine	4.37 ± 0.11	1.05 ± 0.09	3.26 ± 0.25	0.82 ± 0.01
Arginine	ND	0.45 ± 0.01	0.97 ± 0.04	0.88 ± 0.01
Asparagine	2.00 ± 0.04	ND	ND	ND
Aspartate	1.49 ± 0.04	0.50 ± 0.06	2.75 ± 0.12	1.16 ± 0.03
Glutamate	7.07 ± 0.03	ND	3.76 ± 0.26	2.02 ± 0.13
Glutamine	10.38 ± 0.44	ND	ND	ND
Glycine	ND	ND	1.93 ± 0.04	0.47 ± 0.04
Proline	ND	ND	ND	0.69 ± 0.02
Serine	ND	0.09 ± < 0.10	2.73 ± 0.20	0.69 ± 0.02
Tyrosine	1.67 ± 0.05	ND	2.15 ± 0.10	0.32 ± 0.01

^1^ ND: Not Detected.

**Table 3:** AAs concentrations in *A. biennis* mycelium compared to other food sources.

Amino Acid	*A. biennis* Mycelium (mg/g)	Buckwheat *Fagopyrum sagittatum* FAO (mg/g)	Potato *Solanum* *tuberosum* FAO (mg/g)	Lentil *Lens* *culinaris* FAO (mg/g)	Palm Kernel *Elaeis* *guineensis* FAO (mg/g)	Lettuce *Lactuca* *sativa* FAO (mg/g)	Avocado *Persea* *armeniaca* FAO (mg/g)
Leucine	1.41 ± 0.02	7.2	1.21	18.47	4.21	0.83	0.76
Isoleucine	1.32 ± 0.02	4.15	0.76	10.45	2.4	0.5	0.47
Valine	2.15 ± 0.03	8.1	0.93	12.11	3.77	0.71	0.63
Histidine	3.08 ± 0.06	2.6	0.3	6.62	1.47	0.21	0.25
Lysine	4.04 ± 0.45	4.6	0.96	17.39	2.46	0.5	0.59
Methionine	ND ^1^	1.8	0.26	1.94	1.64	0.24	0.29
Phenylalanine	1.27 ± 0.03	4.6	0.8	12.66	2.56	0.67	0.48
Threonine	1.99 ± 0.07	4.4	0.75	9.6	2.22	0.54	0.4
Tryptophan	0.16 ± 0.03	ND	ND	ND	ND	ND	ND
Alanine	4.37 ± 0.11	5.7	0.89	10.41	2.83	0.56	0.82
Arginine	ND	11.96	1	21.01	9.33	0.59	0.47
Asparagine	2 ± 0.04	ND	ND	ND	ND	ND	ND
Aspartate	1.49 ± 0.04	10.8	2.48	27.98	5.75	1.51	3.11
Glutamate	7.07 ± 0.03	2.9	0.12	2.21	1.23	ND	ND
Glutamine	10.38 ± 0.44	21.14	2.04	40.13	11.99	1.34	1.69
Glycine	ND	ND	ND	ND	ND	ND	ND
Proline	ND	7.8	0.76	10.22	3.13	0.54	0.55
Serine	ND	5.3	0.75	10.33	2.29	0.68	0.54
Tyrosine	1.67 ± 0.05	6.1	0.83	12.73	3.28	0.43	0.56

^1^ ND: Not Detected.

## Data Availability

The data used to support the findings of this study can be made available by the corresponding author upon request.
